# Evaluation of a digital health system (PAHcare™) for routine care of patients with pulmonary arterial hypertension: The CBS-PAH study protocol

**DOI:** 10.3389/fpubh.2022.954487

**Published:** 2022-12-07

**Authors:** Gregorio Pérez Peñate, Nuria Ochoa Parra, Juan Antonio Domingo Morera, Amaya Martínez Meñaca, Marta López Ramón, Sergio Cadenas Menéndez, Fernando León Marrero, Sara Gómara de la Cal, Cristina Ghadban Garrido, Patricia Royo Tolosana, Javier Martin Puentes, Rebeca Aldonza Aguayo, Hadis Mahdavi, Gabriela Bacchini Jeanneret, Pilar Escribano Subías

**Affiliations:** ^1^Unidad Multidisciplinar de Circulación Pulmonar, Servicio de Neumología, Hospital Universitario de Gran Canaria Doctor Negrín, Las Palmas de Gran Canaria, Spain; ^2^Unidad Multidisciplinar de Hipertensión Pulmonar, Servicio de Cardiología, Hospital Universitario 12 de Octubre, Madrid, Spain; ^3^Instituto de Investigación Sanitaria Hospital 12 de Octubre (i+12), Madrid, Spain; ^4^Servicio de Neumología, Hospital Universitario Miguel Servet, Zaragoza, Spain; ^5^Servicio de Neumología, Hospital Universitario Marqués de Valdecilla, Santander, Spain; ^6^Servicio de Cardiología, Hospital Universitario Miguel Servet, Zaragoza, Spain; ^7^Servicios de Neumología y Cardiología, Unidad de Hipertensión Pulmonar, Complejo Asistencial Universitario de Salamanca, Salamanca, Spain; ^8^Clinical Research Department, Ferrer, Barcelona, Spain; ^9^Digital Health and Technology, Ferrer, Barcelona, Spain; ^10^Corporate Medical Department, Ferrer, Barcelona, Spain; ^11^Centro de Investigación Biomédica en Red Enfermedades Cardiovasculares (CIBERCV), Madrid, Spain

**Keywords:** pulmonary arterial hypertension, digital intervention, mobile Health, electronic patient-reported outcome, health services research, quality of life, trial, protocol

## Abstract

**Introduction:**

Pulmonary arterial hypertension (PAH) is a rare, multifactorial, chronic condition that requires ongoing monitoring and assessment. PAHcare™ is a novel, patient-centered digital platform that provides software intended for use on patients' mobile phones (downloadable application) and web-based dashboards for use by physicians and health coaches (HC). We describe herein the protocol of a clinical study aimed at evaluating the clinical benefit and safety of PAHcare™ for the routine management of patients with PAH.

**Methods and analysis:**

In this prospective, single cohort, multicenter study, 50 patients with PAH will be recruited at six specialized PAH units from reference hospitals of the public Spanish healthcare system. The PAHcare™ digital health platform allows patients to log health and lifestyle information while also providing structured content for patient education, medication reminders, and behavioral and lifestyle coaching from a remote HC. Evaluation will be primarily focused on the impact of the platform use on the patient's health-related quality of life (HRQoL) *via* questionnaires completion through electronic patient-reported outcomes. Moreover, the analysis of the impact on the patient's functional status, signs and symptoms of PAH, patient costs and healthcare resource utilization, satisfaction, knowledge of the disease and its management, and adherence to and safety of the platform will be secondary outcomes. The clinical investigation started in July 2021 and is expected to end by September 2022.

**Discussion:**

The PAHcare™ platform is anticipated to provide direct benefits to healthcare professionals, patients, and caregivers. These include the simplification of the multidisciplinary approach needed to tailor routine PAH management, enhancement of the patient/healthcare professional interaction, patient's empowerment to become more actively involved in the management and treatment of the disease, and increase of the patient's and caregiver's knowledge on PAH.

## Introduction

Pulmonary arterial hypertension (PAH) is a rare, multifactorial, chronic condition characterized by a progressive and sustained narrowing of the pulmonary vessels that leads to elevated mean pulmonary artery pressure and may eventually result in right ventricular failure and premature death (1). PAH includes different forms with virtually identical pathological changes of the lung microcirculation that share a similar clinical picture, with dyspnea, fatigue, chest pain, syncope, and peripheral oedema (1).

Although there has been significant progress in the available pharmacological treatments to alleviate disease symptoms and slow the disease progression in the past two decades, PAH has a devastating impact not only on the patient's physical daily activity but in several dimensions of health-related quality of life (HRQoL) (2–4). Indeed, different surveys conducted worldwide have consistently shown that most PAH patients report that between a quarter and half of them suffer from depression and anxiety symptoms; between half and three quarters are unable to work -with the accompanying reduction in average household income-; and a significant proportion report feelings of social isolation, reduced social activity, or disrupted sexual relationships, among other complaints (2–4). Of note, although PAH-targeted pharmacological therapies improve HRQoL, especially for the physical domains, QoL impairment may persist despite optimal treatment (5, 6). The results of these surveys support the need for multidisciplinary PAH management that should include healthcare providers (HCPs) and caregivers and take into account the patient's perspective (7, 8). Besides, PAH management requires an active, regular, and intense partnership between patients and HCPs that needs to be continued in-between visits to monitor medication adherence, identify and manage symptoms and adverse events, recommend self-lifestyle interventions, etc. (1). The overall goal of the proposed holistic approach is to enhance the patient's engagement and self-management through effective education and the delivery and communication of timely, high-quality information able to assess and improve all aspects of wellbeing for both patients and caregivers (2, 3, 9). In this context, the use of digital interventions [electronic health (eHealth), or mobile Health (mHealth) when referred to mobile and wireless technologies to support the achievement of health objectives] that facilitate the management and delivery of healthcare has been shown to have a beneficial impact in chronic disease management (10). For instance, mHealth interventions–most frequently text messaging to send reminders, alerts, education, motivation, and prevention- have been reported to improve symptoms of asthma, chronic pulmonary disease, or heart failure; glycemic control in diabetes patients; blood pressure in hypertensive patients; attendance rates, or improved adherence to tuberculosis and human immunodeficiency virus therapy (10).

In the particular case of PAH, a recent review of the literature evaluating the use of eHealth strategies found that they were safe, accurate, and reliable, contributing to the clinical management of PAH patients (11). However, only in two of the studies, the strategy was part of the medical management, and most participants pertained to World Health Organization (WHO) classification Group 2 (post-capillary PAH), which has completely different health management needs than the WHO Group 1 PH (pre-capillary PAH) (11). Lastly, none of the studies assessed the impact of eHealth strategies on the patient's HRQoL (11).

Considering that there is an increased recognition that the management of PAH must aim to reduce the impact of the disease in HRQoL (9), it has been suggested that it should be assessed through PAH-specific patient-reported outcomes measures (PROs) (3). PROs, which are a form of validated self-report instruments which use patients' views to assess their health status and wellbeing (12), can be systematically and electronically collected (ePROs) through eHealth technologies such as tablets, mobile phones, or web-based platforms (13, 14). A recent review of the literature on the use of ePROs in routine clinical practice for the management of chronic diseases reported that they may improve patient survival (in patients with cancer), lead to more efficient utilization of healthcare resources in different chronic conditions (e.g., epilepsy, coronary heart disease, narcolepsy, sleep apnea, or kidney failure), improve symptom management and individualized care compared with usual care (e.g., in patients undergoing radiotherapy or chemotherapy), improve treatment adherence and monitoring (in patients on oral chemotherapy), and reduced the risk of SARS-CoV-2 virus transmission during the COVID-19 pandemics (14). Besides ePROs assisting clinicians with early detection of complications, immediate action, and potentially reducing symptom burden, complications, and readmissions to the hospital, their use empowers patients and improves patient-clinician communication (13). Last but not least, the routine collection of ePROs may complement clinical evaluations by assessing the patients' perspectives on their HRQoL, unmet needs and concerns, and care priorities (14).

In this paper, we describe the protocol of the clinical benefit and safety (CBS)-PAH clinical investigation, an observational, multicenter study aimed at assessing the safety and performance of the PAHcare™ digital platform in patients with PAH. This platform is based on the Wellthy CARE™ (WC) digital intervention platform (DIP), consisting of a mobile application for smartphones, a web portal for health coaches to visualize patient data, make data entry if needed and communicate with patients and a web platform for physicians to visualize patient data. The performance of the WC DIP has been previously studied in the real-world setting in patients with chronic diseases such as type 2 diabetes (T2D) and hypertension (15–18). In patients with T2D, the use of a 16-week structured self-management program delivered with the WC mobile application showed improved glycemic control, weight reduction, and self-management (15, 16). In patients with T2D and hypertension previously using WC for their diabetes management, the addition of hypertension self-management education and self-reported blood pressure (BP) measures showed that the platform facilitated glycemic control while achieving a significant reduction in BP (17). Lastly, a study assessing the WC platform performance during the COVID-19 lockdown showed similar decreases in average blood glucose to those observed in the pre-lockdown despite increased ePROs (18). The results confirmed that the use of ePROs was acceptable to patients as a supplement to the usual care at the physician's office, enabling patients to maintain continuity of care (18).

The CBS-PAH study will assess the performance of the PAHcare™ platform for patients with PAH during 6 months through the key components of the patient-facing mobile app, namely ePROs, educational material, human health coach support through chats and calls, and additional professional psychological support through an external portal.

## Methods and analysis

### Study design

This is a prospective, single cohort, multicenter study enrolling patients with PAH at five specialized PAH units (six investigators) from reference hospitals of the public Spanish healthcare system (Supplementary Table 1).

The methodological approach of the study involves the use of a digital health platform (PAHcare™) intended for both patients with PAH and their care team for 6 months. The platform consists of a patient-facing mobile application (Android, iOS) and dashboards for physicians and health coaches (HCs). The application allows patients to log various clinical and laboratory parameters while also providing evidence-based patient education and health coaching from a remote HC. Patients will be able to conveniently self-report *via* the PAHcare™ mobile application using ePROs as outcome measures.

PAH is a rare disease, and, given the anticipated low number of patients eligible for this clinical investigation, the inclusion of a control group (i.e., PAH patients not using the platform) was not considered. Furthermore, the PAHcare™ platform is an approved Class I medical device (MD) that will be available to all PAH patients and therefore, the inclusion of a control group (i.e., PAH patients not using the device) would require consent from patients to prevent them from accessing and using the platform. Moreover, allocating the anticipated low number of eligible patients into two arms (using and not using PAHcare™) would have further decreased the number of patients for analysis and, therefore, the statistical power.

### Objectives and outcome measures

The main aim of the CBS-PAH study is to evaluate the clinical benefit and safety of use of the PAHcare™ platform as a novel, patient-centered intervention for patients with PAH.

#### Primary objective

The main objective is to determine whether the PAHcare™ intervention can lead to improved disease burden, namely HRQoL, as evidenced by scores in Spanish validated versions of the General Questionnaire and the Euroqol 5-dimension questionnaire (EQ-5D-5L) (19, 20), and the emPHasis-10, a short questionnaire developed to specifically assess HRQoL in PAH (21, 22).

#### Secondary objectives

- To determine the impact of the PAHcare™ intervention on the clinical evolution of the disease, namely whether it can lead to the following outcomes in patients with PAH:
  ▸ Improvement (change) in WHO functional classification (WHO FC) of PAH–which grades the impact of the disease in the patient's physical activity (Supplementary Table 2) between 6 months before inclusion in the clinical investigation and throughout the follow-up period.
  ▸ Improvement (change) in the 6-min walk test (6 MWT) or cardiopulmonary exercise test (CPET) between 6 months before inclusion in the clinical investigation and throughout the follow-up period. The 6MWT is a sub-maximal exercise test that assesses aerobic capacity and endurance to evaluate PAH severity and progression (23). The CPET is a more complete test that involves measurements of respiratory oxygen uptake, carbon dioxide production, and ventilatory measures during a symptom-limited exercise test (24). It is used to estimate the severity of the disease and assesses the patient's response to therapy.
  ▸ Improvement (change) in scores of a 100-mm graded visual analog scale (VAS) self-rating perception of PAH symptoms between baseline and final visit.
  ▸ Improvement (change) in the incidence, frequency, and intensity of PAH signs and symptoms (i.e., dyspnea, orthopnea, fatigue, syncope, chest pain, and oedema) between 6 months before inclusion in the clinical investigation and throughout the follow-up period.

- To evaluate the impact of the PAHcare™ intervention on disease-associated costs and, indirectly, on the clinical evolution of PAH through:
  ▸ Changes in the number of PAH-associated hospital admissions (including emergency room and unscheduled visits/consultations) during the 6 months before inclusion in the clinical investigation and during the clinical investigation period.
  ▸ Description of the reason for PAH-associated hospitalization/visit/emergency room admissions, duration of hospitalization, and procedures during the 6 months before inclusion in the clinical investigation and during the clinical investigation period.
  ▸ Description of healthcare, formal and informal care costs associated with PAH and changes between baseline and final visit, assessed using an *ad-hoc* questionnaire (Supplementary Table 3).
  • To evaluate the patients' reported satisfaction with the PAHcare™ use, measured by the 8-item Client Satisfaction Questionnaire (CSQ-8) after 6 months of use (25).
  • To evaluate the consumption of educational contents through the percentage of course completion along the structured educational pathway available in the platform.
  • To evaluate patients' adherence to the platform by describing platform interactions and month-wise changes in the number of interactions, including number and duration of logs (ePROs), content consumed (magazines, lessons, quizzes), and number and duration of HC calls and HC chats completed.
  • To determine whether the PAHcare™ mobile application is safe through the number of patients who experience a device incident during the clinical investigation period.

### Participants and recruitment

Patients (*n* = 50) will be recruited from five clinical centers across Spain (Supplementary Table 1). All PAH patients who are routinely followed up for disease monitoring in the participant sites will be considered for eligibility to participate in the CBS-PAH study. Considering the lower prevalence of PAH with more severe disease (WHO FC III/IV) compared to those with less severe disease (WHO FC I/II), investigators will be allowed to pre-screen patients according to their disease severity to ensure representation of I/II and III/IV classes. Nevertheless, the recruitment priority will be to reach the planned number of study patients. The recruitment of patients for this clinical investigation will be competitive. Even though the distribution of enrolment among the participating sites cannot be anticipated, PAH units covering larger areas and consequently following more patients are more likely to recruit higher numbers of patients.

The inclusion and exclusion criteria are summarized in Table 1. The patient recruitment started in July 2021, and it is planned to end by March 2022.

**Table 1:** Inclusion and exclusion criteria for trial participation.

**Inclusion criteria**
• Age ≥18 years
• Diagnosed with PAH (Pulmonary Hypertension WHO Group 1) of any WHO Functional Class (I to IV)
• Deemed fit for self-care/caregiver-driven program for PAH by the treating physician
• Signed informed consent
• Able to read, speak, or understand Spanish
**Exclusion criteria**
• If women, pregnant, lactating, or planning a pregnancy in the next 6 months
• Patients who have undergone a major surgical intervention within 30 days before inclusion in the clinical investigation or suffering from complications that may hinder the complete utilization of the patient support program
• Hearing disability and not using hearing aids
• Visually impaired
• With intellectual disabilities
• Patients who have not access to a smartphone

WHO, World Health Organization.

### Clinical investigation procedures

**Figure 1:**
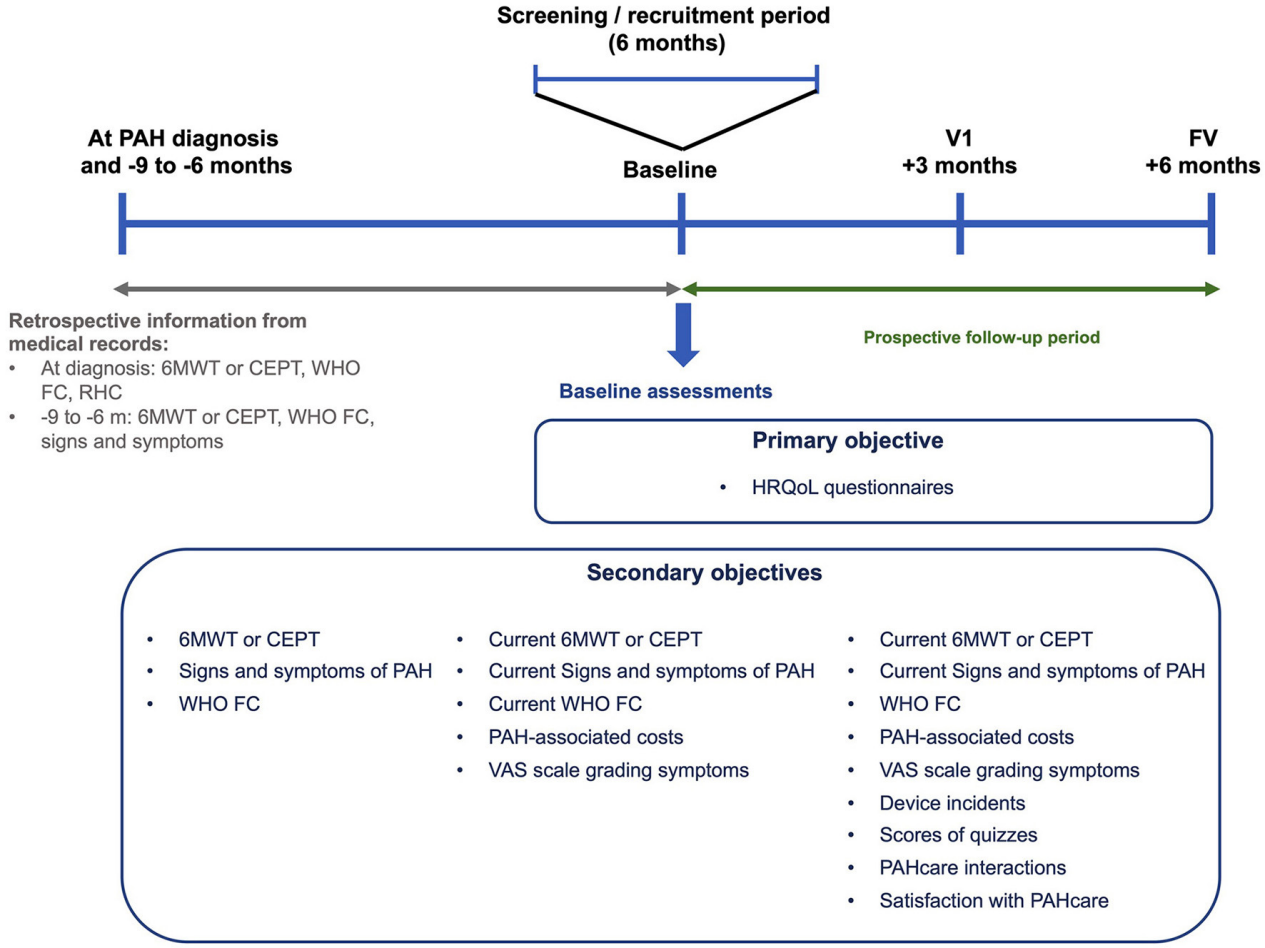
Diagram summarizing the clinical investigation design and main assessments. 6 MWT, 6-minute walking test; CEPT, cardiopulmonary exercise test; FV, final visit; PAH, pulmonary arterial hypertension; HRQoL, health-related quality of life; RHC, right heart catheterization; V1, visit 1; VAS, visual analog scale; WHO FC, World Health Organization functional class.

The CBS-PAH study schedule and procedures are presented in Figure 1 and Table 2. The intervention consists of the use of the PAHcare™ digital health platform for 6 months. To avoid interferences with routine clinical practice, all clinical investigation visits have been foreseen that match the clinical guidelines for PAH patients in Spain, which recommend scheduling monitoring visits every 3–6 months (26). Likewise, assessments will be those routinely performed in routine practice except for the HRQoL, satisfaction, and costs questionnaires.

**Table 2:** CBS-PAH study schedule of enrolment, intervention, and assessment.

	**Study procedures**			
	**Visit (eCRF/ePRO website)**			**App log (platform database)**
**Timepoint**	**Baseline**	**V1 3 m**	**FV 6 m**	*t_x_* ^*^
Eligibility screen	X			
Informed consent	X			
**Intervention**				
*PAHCare*™	•———————————————————————————•			
**Assessments**				
App configuration with the HC's help				X
Demographic characteristics				X
6 MWT or CEPT, WHO FC, and RHC at diagnosis	X			
6 MWT or CEPT, WHO FC, signs and symptoms (−9 to −6m, retrospective)	X			
Current WHO FC^a^	X		X	
Current 6 MWT or CEPT^a^	X		X	
HRQoL questionnaires (emPHasis-10 and EQ-5D-5L)	X	X	X	
Signs and symptoms of PAH	X	X	X	
Patient's signs and symptoms perception (VAS)	X	X	X	
Patients' satisfaction with PAHcare™ (CSQ-8)			X	
Device incidents		X	X	X^+^
Disease-associated costs questionnaire	X		X	
Hospital and emergency room admissions and unscheduled visits/consultations^b^	X		X	X
Scores of quizzes assessing disease knowledge				X
App interactions				X

* As scheduled by the application.

+ Not directly through the app, all device incidents reported by patients via HC or directly by the HC will be documented in compliance with applied regulations and guidelines of ISO13485.

a Routine assessment (one or the other) that will be mandatory at the indicated clinical investigation visits.

b Assessment included in the disease-associated costs questionnaire.

6MWT, 6-minute walking test; App, application; CEPT, cardiopulmonary exercise test; eCRF, electronic case report form; ePRO, electronic patient reported outcome measure; FV, final visit at 6 months; m, months; HC, health coach; PAH, pulmonary arterial hypertension; HRQoL, health-related quality of life; RHC, right heart catheterization; V1, visit 1 at 3 months; VAS, visual analog scale; WHO FC, World Health Organization functional class.

### PAHcare™ digital health platform description

The PAHcare™ digital platform is a patient support program for adult patients with PAH that was developed by Ferrer Internacional S.A. with the technical support of Wellthy Therapeutics. This medical device is intended to provide a series of digital tools to aid in managing the disease and facilitate alleviation through behavioral and lifestyle coaching, structured content support, and medication reminders.

**Figure 2:**
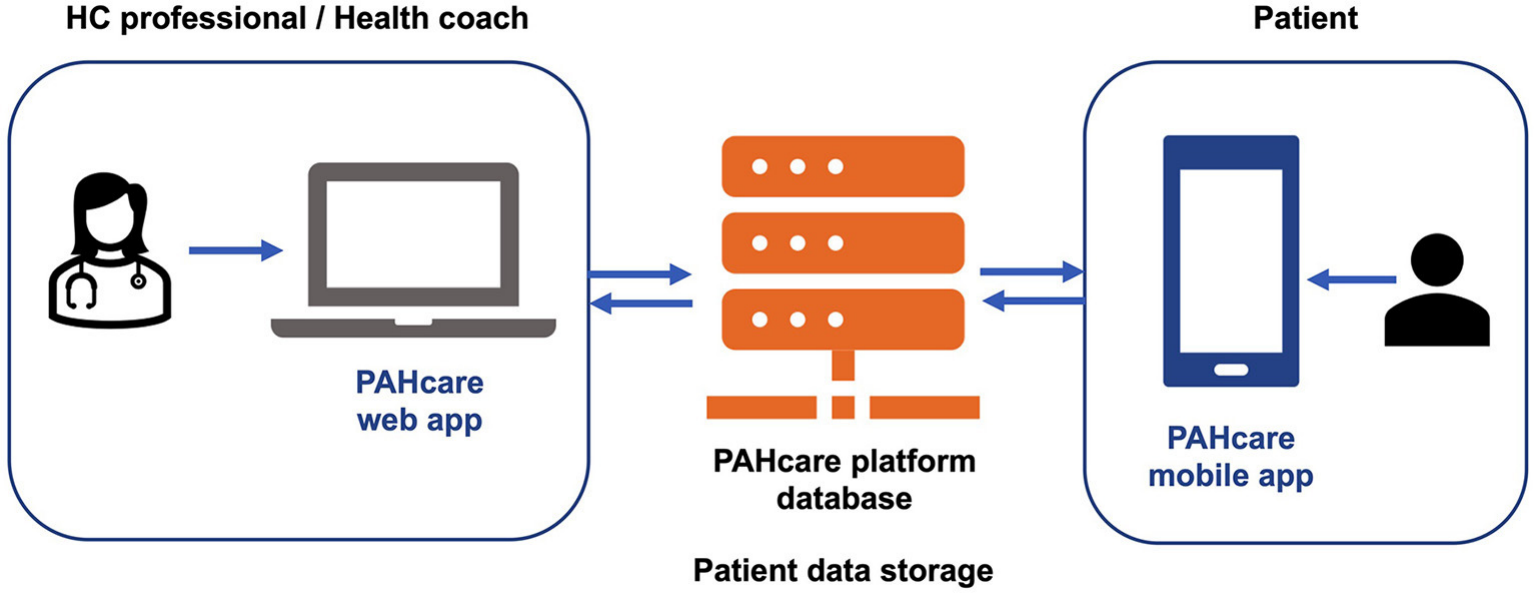
Schematic representation of the PAHcare™ digital platform.

PAHcare™ is proposed to be used by the patients, physicians, and health coaches (HCs) who participate in the CBS-PAH study (Figure 2). From the patient's perspective, the functionalities are offered *via* the PAHcare™ mobile application installed on his/her personal smartphone. Patients who fulfill eligibility criteria and accept to participate in the study will be asked to download PAHcare™ through the App Store (iOS) or Play Store (Android), will be provided with a quick response (QR) code to activate the application, and will be assigned a specialized HC according to their prescribed treatment. Dashboards for platform use by healthcare professionals will be installed in the computers of the participating centers and the laptops provided to the HCs (Figure 2). That way, the patient's clinical information and logs will be delivered to the physicians and HCs *vi*a the PAHcare™ web application, their main interface to the system.

The permissions to add, edit, and view data captured in the platform are shown in Table 3, and the patient's medical history data requested by the application, logged in by the patient or HCs, is shown in Table 4.

**Table 3:** Permissions to add, edit, and view data captured in the PAHcare digital platform.

**Data captured in the platform**	**User**	**Physician**	**Health coach**
Basic personal information	Add View	View	Add View
Medical history	Add View	View	Add View
Lifestyle information^*^	Add View	View	Add View
6 MWT/CEPT	View	View	Add View
Laboratory data	View	View	Add View

6 MWT, 6-minute walking test; CEPT, cardiopulmonary exercise test.

* Including activity, calories, weight, and water.

**Table 4:** Medical history data requested by the PAHcare™ application.

**Primary categories**	**Parent sub-categories**	**Child sub-categories**	**Options for filling**
Current conditions	PAH Data	PAH Subtype	• Idiopathic • Heritable • Other
		If Other (if applicable, i.e., NOT mandatory)	Free text
		Current WHO FC	• I • II • III • IV
	Comorbidities (Ability to add multiple comorbidities as required by a patient)	Date of diagnosis	MM/YYYY
		Condition name	Free text
		Date of diagnosis	MM/YYYY
Previous conditions (Ability to add multiple previous conditions as required by a patient)	Condition name	N/A	Free text
	Date of diagnosis (if applicable, i.e., NOT mandatory)	N/A	MM/YYYY
	End date (if applicable, i.e., NOT mandatory)	N/A	MM/YYYY
Allergies (Ability to add multiple allergies as required by a patient)	N/A	N/A	Free-Text
General symptoms (Ability to add multiple entries as required by a patient)	N/A	N/A	Free-Text

PAH, pulmonary arterial hypertension; WHO FC, World Health Organization functional class.

The patient-facing mobile app PAHcare™ has the following key components:

1. PROs data, including logging of symptoms, adverse events (only available for patients on Ferrer's medications), hospitalizations, medications, clinical and laboratory parameters, and lifestyle information including activity and meals.
2. PAH-related, evidence-backed content as magazine articles, Frequently Asked Questions (FAQs), lessons, and quizzes in textual and rich media formats.
3. Human HC support through chats and calls through either of the two following modalities:
  a. A specially trained HC for patients treated with the prostacyclin analog treprostinil. This HC will give support as needed with the treatment and associated medical devices for the administration of treprostinil (e.g., infusion pump or catheter). In addition, the HC will help in the reporting and management of adverse events and give support for the digital platform and mobile application, self-monitoring, lifestyle interventions, behavioral modifications, and symptom management.
  b. An HC for all other patients receiving treatments other than treprostinil, focusing on support for the digital platform and mobile application, self-monitoring, lifestyle interventions, behavioral modifications, and symptom management.

4. Additional professional psychological support through an external portal (only available for patients on Ferrer's medications), as needed.
5. Other components of the platform involve a brief snapshot of the patient-reported data summarized for use by the HCPs and the study personnel, as well as caregiver support available through SMS and calls for all patients using the app.

Access to the PAHcare™ digital health platform will be granted to the patients for 6 months of continuous use right after their enrolment in the study. Physicians and HCPs will have access to the system until the end of the trial. The patients will be informed that the PAHcare™ system is not intended to be and does not constitute a substitute for professional medical advice, that is not meant to be used as an emergency service and urgent issues need to be consulted with the treating physician and medical team.

### Data collection and analysis

#### Data collection

Data collection will be undertaken by the PAHcare™ digital platform, which consists of three modules (Figure 2): (1) the PAHcare™ mobile app, which will be used by the patients either spontaneously or periodically, (2) the PAHcare™ web portal, which will be used by the physicians and HCs participating in the study to monitor and review the collected patient data periodically, as well as by the HCs to register patient's data such as treatment plan, clinical evolution, etc., and (3) the PAHcare™ platform storage module and database.

#### Types of data

The data from the clinical investigation will be recorded from different sources (see also Table 2):

- *Medical records/study source documents*. Data already collected in medical records and study source documents or collected during clinical investigation visits (i.e., baseline, at 3 months, and at 6 months) will be registered in an electronic Case Report Form (eCRF). Data will include demographic characteristics, medical history and treatments, WHO functional classification, presence, frequency (times/day and/or week), and intensity (mild, moderate, or severe) of PAH signs and symptoms (dyspnoea, orthopnoea, fatigue, syncope, chest pain, and oedema), 6 MWT or CEPT score, and patient's PAH symptoms perception graded on a VAS scale.
- *PAHcare*™ platform database, which will collect and record data logged by patients regarding, hospital and emergency room admissions and unscheduled visits/consultations to the PAH unit, progress of patients through structured educational contents and knowledge of the disease and its management, and platform interactions [i.e., number of logs (PROs), content consumed (magazines, lessons, quizzes), and HC calls and chats completed]. Device incidents reported by patients *via* HC or directly by the HC will be collected and documented in compliance with applied regulations and guidelines of ISO13485.
- *ePROs website*, accessible *via* a link that will be sent to patients' mobile phones, which will collect data recorded during study visits regarding the HRQoL, patient's satisfaction questionnaires and disease-associated healthcare resource use and costs questionnaire.

#### Data security

The processing of the data to be compiled during the study will be performed in accordance with Spanish Organic Law 3/2018, which develops the General Data Protection Regulation 2016/679 on data protection and privacy for all individuals within the European Union (27).

The data protection security measures include that: (1) each patient will be informed before the clinical investigation starts about how his/her personal data will be processed. The investigator and the Ferrer Internacional will guarantee its pseudonymization and compliance with data protection law; (2) that the data for the study collected using the PAHcare™ platform, ePRO website, and eCRF will be assigned an identification code, available only to the investigation team/staff; (3) that the access to personal information will be restricted to the investigators and collaborators, competent national authorities, ethics committee, and monitoring and auditing teams, who will ensure confidentiality according to the abovementioned Spanish and European personal data regulations.

#### Data monitoring

Investigators and institutions will allow the monitoring and audits by the Health Authorities or Ferrer Internacional, giving direct access to data and original source documents. Following applicable regulations and Good Clinical Practice (GCP), the monitor will regularly visit or contact the participating sites to monitor and evaluate the progress of the clinical investigation; examine the data collected; carry out a verification of the source documents; and identify any problems and find solutions. Moreover, data recorded in the PAHcare™ platform will be examined by Ferrer Internacional and shared with the monitor every month.

### Sample size calculation

Previous references for the primary endpoint (i.e., changes in HRQoL) in population with PAH are unavailable, precluding a formal calculation of the required sample size. Considering the low prevalence of PAH and the pilot nature of this study, a sample of 50 patients was deemed sufficient to give information on changes in questionnaire scores.

#### Data analysis plan

The primary and secondary objectives will be carried out in two population sets: (1) the full analysis set (FAS), defined as all selected patients, and (2) evaluable population (EP), defined as all selected patients with data available to analyze the primary endpoints. All patients with available data for safety and patient-reported outcomes will be considered to analyze the corresponding endpoints.

Data registered in two different databases, such as hospital and emergency room admissions and unscheduled visits/consultations, collected in the ePROs and the platform database, will be inspected for consistency. In case of discrepancies, data collected in the ePROs will prevail for the analysis.

The analysis of this clinical investigation is descriptive and is not based on testing formal null hypotheses. The analyses will be focused on the description of clinical variables during different time points throughout the follow-up period, namely at baseline (BL), at Visit 1 (V; 3 months after the BL visit, and at the final visit (FV; FV, 6 months after the BL visit. For this, categorical variables will be summarized through frequency tables (absolute and relative) and continuous variables through measures of central tendency and dispersion (i.e., mean, standard deviation, minimum, maximum, median, and quartiles).

The analyses will aim to evaluate the changes in the outcome measures over time, and changes in variables at baseline and the different clinical investigation visits (V1, when applicable, and FV) will be analyzed using statistical methods for paired data comparisons. Continuous variables will be compared using the parametric paired *t*-test and its non-parametric counterpart, the Wilcoxon test, and categorical variables will be compared using McNemar's test. The significance threshold will be set at a two-sided =0.05. This clinical investigation does not foresee the application of methods for imputation of missing data.

### Safety reporting

The PAHcare™ platform is a digital device and, as software, no safety issues are foreseen. Moreover, this study does not intend to evaluate a commercial medicinal product, and all treatments received by patients, are marketed medicines with their corresponding pharmacovigilance mechanisms in place. Therefore, adverse drug reactions (ADRs) and other safety information occurring during the study investigation will be reported through pharmacovigilance mechanisms.

The study will assess the safety of the PAHcare™ platform, measuring the occurrence of any device serious adverse event (SAE) or medical device incidents (MDI) will be notified through pharmacovigilance and reporting mechanisms. A medical device incident will be defined as any malfunction or deterioration in the characteristics or performance of the device, including use error due to ergonomic features, any inadequacy in the information supplied by the manufacturer, and any undesirable side-effect. Moreover, all suspected MDIs associated with the medical devices used for administering the prostacyclin analog treprostinil (such as, but not limited to, the infusion pump or the catheter) will be reported by the investigator to the Ferrer Internacional Corporate Pharmacovigilance Department.

### Ethics and dissemination

The Clinical Investigation Plan (V4.0 dated 9 March 2022) has received ethical approval from the ethics committee of the Hospital 12 de Octubre in Madrid (22^nd^ March 2022; registry number 21/298). Any substantial modification affecting criteria for selecting the population, response variables, procedures, or data analysis, or any other modification considered as substantial for any other reason, will be presented to the Ethics Committee for approval before its implementation.

The PAHcare™ medical device assessed in this clinical investigation has been approved in Europe as a Class I investigation medical device as per Medical Devices Directive (93/42/EEC) (28) for its use by PAH patients (26 March 2021, reference number 1291298).

The clinical investigation was registered at the NEOPS database for clinical investigation studies with medical devices approved in the EU of the Spanish Agency of Medicines and Medical Devices (AEMPS; https://neops.aemps.es/neops/login) on 6 June 2021 (registry number 21-0050). Recruitment started in Summer 2021. We expect to recruit the whole sample by the end of March.

This study will be conducted in accordance with the Declaration of Helsinki of 1975 (in its most recently amended version) and international standard ISO 14155 “Good Clinical Practice” guidelines. Moreover, this clinical investigation will be conducted in accordance with the European and Spanish Regulations on Medical Devices (29, 30). The patients/participants will provide written informed consent to participate in this study.

The study findings will be published in peer-reviewed scientific journals and presentations in scientific meetings. Summaries of the results will also be made available to investigators for dissemination within their clinics.

## Discussion

The adoption of eHealth technologies has shown to be useful regarding strategies aimed at monitoring patients with PAH (11). However, the interventions reported to date were mainly focused on remote disease monitoring using devices such as a digital pedometer, real-time wireless monitoring of heart rate, cardiac index and cardiac output, 6-min walk home test applications, step oximetry linked to a computer, or an implantable hemodynamic monitor of drug-induced variations in positive airway pressure (11). Besides, in most cases, these devices were not integrated into the patient's medical management, and none of the studies assessed the impact of the intervention in important clinical outcomes such as changes in HCPs visits, mortality, hospitalizations, and none evaluated its impact on HRQoL (11). The CBS-PAH clinical investigation described herein seeks to evaluate the acceptability and usefulness of the PAHcare™ digital platform to reduce PAH burden, including patients' emotional wellbeing.

The PAHcare™ platform is anticipated to provide direct benefits to HCPs, patients, and caregivers. From the HCPs' perspective, PAHcare™ is expected to simplify the multidisciplinary approach needed to tailor routine PAH management, a chronic condition requiring ongoing monitoring and assessment. Besides, it should lead to more efficient use of health resources through the opportunity to optimize therapy or track adverse events. Moreover, the quantitative HRQoL assessment through the digital platform will enhance the patient/HCP interaction. This will help HCPs better understand the patients' individual needs other than those purely clinical ones, such as their experience with the disease in terms of functional burden and psychosocial impact. Lastly, since the PAHcare™ will be publicly launched for all PAH patients in the future, an added benefit of its implementation in routine care may be the generation of prospective, longitudinal, real-world data that may yield valuable insights into PAH epidemiology, progression, and patient's wellbeing, among others. From the patient's and caregiver's perspective, the digital platform can provide access to medical care in areas where such services may be limited, not regularly provided, or difficult to reach if the patient has restricted mobility or fatigue. Most importantly, it will empower patients to become more actively involved in managing and treating their disease, which has been shown to improve a range of outcomes in patients with PAH, including psychosocial wellbeing (7). Finally, the delivery of specific information and education on the disease from a high-quality source will help patients and caregivers understand the complexity and challenges of the disease and its treatment and show them how it will impact their physical and emotional daily lives (7).

To our knowledge, this is the first study that will examine the effects of using mHealth strategies in patients with PAH. However, the study has limitations that must be acknowledged. Firstly, the clinical investigation design lacking a control arm (i.e., patients not using the platform) prevents the direct assessment of platform use effects. However, the retrospective assessment of clinical variables, including 6 MWT or CEPT, WHO-FC, and PAH symptoms and signs, may partially compensate for this limitation. Secondly, the prevalence of patients with marked or severe activity limitation (III/IV WHO FC) is low, while most patients without limitation of activity (I WHO FC) remain undiagnosed due to their mild symptoms. Thus, we anticipate that the clinical investigation population is likely to be biased toward patients with WHO FC II. To avoid this bias, investigators will be encouraged to pre-screen patients to balance the representation of less (I/II) and more (III/IV) severe WHO FCs. Other limitations of the study are associated with the digital nature of the platform, such as loss of data entered by the users, user interface errors, functionalities not working, or failure to access patient data. Additionally, non-adherence to the digital intervention, incorrect usage of the platform, and discontinuation of the intervention may interfere with the outcomes and interpretation of the results.

In summary, the PAHcare™ digital platform will offer the opportunity to facilitate PAH self-management by providing continuity-of-care, encouraging patients' engagement, and promoting self-care behavior through educational content, lifestyle recommendations, patient data storage, and support of a remote HC.

## Ethics statement

The competing Ethics Committee of the Hospital 12 de Octubre, Madrid, Spain, approved the study protocol V4.0 March 22, 2022 (FPAH-CI-2101).

## Author contributions

PE and RA contributed to conception, design, and planned analyses of the study. GB contributed to conception of the study and to draft manuscript preparation. HM contributed to planned analyses of the study. PE, JD, ML, GP, AM, SC, NO, SG, PR, FL, CG, and JM will be committed to recruitment during the study duration. All authors approved the protocol, critically revised the manuscript and gave their final approval.
