# Supplementary material for: Evaluation of a digital health system (PAHcare™) for routine care of patients with pulmonary arterial hypertension: The CBS-PAH study protocol
Source: Front Public Health. 2022 Dec 7;10:954487. doi: 10.3389/fpubh.2022.954487 (PMC9768478; doi:10.3389/fpubh.2022.954487)
Supplement: Supplementary file 1 [file Table_1.DOCX]

**Supplementary Information**

*The authors have provided these supplemental materials to give the readers additional information*

[Supplementary Table 1. List of participating clinical investigation sites across Spain. 2](#_Toc96854902)

[Supplementary Table 2. Description of who PAH functional class classification 3](#_Toc96854903)

[Supplementary Table 3. DISEASE-RELATED HEALTHCARE COSTS AD HOC QUESTIONNAIRE 4](#_Toc96854902)

# Supplementary Table 1. List of participating clinical investigation sites across Spain.

| Center number | Center name and location |
| --- | --- |
| 01 | Hospital Universitario 12 de Octubre. Madrid, Spain |
| 02 | Hospital Universitario Marqués de Valdecilla. Santander, Spain |
| 03 | Hospital Clínico Universitario de Salamanca. Salamanca, Spain |
| 04 | Hospital Universitario Miguel Servet (Servicio de Neumología). Zaragoza, Spain |
| 05 | Hospital Universitario Miguel Servet (Servicio de Cardiología). Zaragoza, Spain |
| 06 | Hospital Universitario de Gran Canaria Dr. Negrín. Las Palmas de Gran Canaria, Spain |

# Supplementary Table 2. Description of who PAH functional class classification

(available from http://www.who.int/gard)

| Functional class | Description |
| --- | --- |
| I | No limitation of usual physical activity; ordinary physical activity does not cause dyspnea, fatigue, chest pain, or presyncope |
| II | Mild limitation of physical activity; no discomfort at rest; but normal activity causes increased dyspnea, fatigue, chest pain, or presyncope |
| III | Marked limitation of activity; no discomfort at rest but less than normal physical activity causes increased dyspnea, fatigue, chest pain, or presyncope |
| IV | Unable to perform physical activity at rest; may have signs of right ventricle failure; symptoms increased by almost any physical activity |

# SUPPLEMENTARY TABLE 3. Disease-related healthcare costs ad hoc questionnaire

Disclaimer (original Spanish version): The following cost questionnaire is the original version which was included in the study FPAH-CI-2101 (“*Clinical benefit and patient-reported outcomes of the use of PAHcare™ platform for routine care of patients with pulmonary arterial hypertension*”). The sections included in the questionnaire are the following:

- Clinical visits & hospitalisations
- Paramedical treatment visits
- Specialized equipment & devices
- Formal care
- Informal care
- Patient travel expenses
- Employment situation

1. **Half-year questionnaire (From ____.____.____ to ____.____.____) (DD-MM-YYYY)**

| **CLINICAL VISITS & HOSPITALISATIONS** | | | | |
| --- | --- | --- | --- | --- |
| 1. During the last 6 months, due to your disease (PAH), have you had to see a doctor (e.g., general practitioner, cardiologist, etc)? (also telephone calls, apart from the ones for the clinical study) or to make an appointment for a home visit? | | Yes.……………………………  No……..………………………  If you have checked “Yes” please complete the subsequent tables A and B. If you have  checked “No” please move on to question number 2. | | 1  2 |
| Table A. If “Yes”, which type of treatments did you receive? (e.g., lab test, filled prescription)   \| GP and specialist visits (specialization) \| Public or Private (insurance) service? \| Number of contacts/visits during the last 6 months, date (dd-mm-yyyy) of contact/visit and center/hospital \| Which procedures were done during all contacts/visits? \| How many procedures of each class were done in total? \| \| --- \| --- \| --- \| --- \| --- \| \| General practitioner \| □ Public  □ Private \| □ Visit 1 ___.___.___ _________  □ Visit 2 ___.___.___ _________  □ Visit 3 __.___.___ _________  □ Visit 4 ___.___.___ _________  □ Visit 5 ___.___.___ _________  □ Visit 6 ___.___.___ _________  □ Visit 7 ___.___.___ _________  □ Visit 8 ___.___.___ _________  □ Visit 9 ___.___.___ _________  □ Visit 10 ___.___.___ ________ \| □ Blood test  □ Prescription filled  □ Telephone call  □ X-ray  □ Routine medical check-up  □ ___________  □ ___________ \| __________________  __________________  _________  __________________ \| \| Nurse \| □ Public  □ Private \| □ Visit 1 ___.___.___ _________  □ Visit 2 ___.___.___ _________  □ Visit 3 __.___.___ _________  □ Visit 4 ___.___.___ _________  □ Visit 5 ___.___.___ _________  □ Visit 6 ___.___.___ _________  □ Visit 7 ___.___.___ _________  □ Visit 8 ___.___.___ _________  □ Visit 9 ___.___.___ _________  □ Visit 10 ___.___.___ ________  □ Visit 11 ___.___.___ ________  □ Visit 12 ___.___.___ ________ \| □ Blood test  □ Prescription filled  □ Telephone call  □ Training on pump use/disease management  □ Dose titration of PAH treatment  □ 6 min walking test  □ Pump error  □ Pump change  □ Routine check-up  □ ___________ \| __________________  __________________  _________   _________  ____________________________________  _________ \| \| Pulmonologist \| □ Public  □ Private \| □ Visit 1 ___.___.___ _________  □ Visit 2 ___.___.___ _________  □ Visit 3 __.___.___ _________  □ Visit 4 ___.___.___ _________  □ Visit 5 ___.___.___ _________  □ Visit 6 ___.___.___ _________  □ Visit 7 ___.___.___ _________  □ Visit 8 ___.___.___ _________  □ Visit 9 ___.___.___ _________  □ Visit 10 ___.___.___ ________  □ Visit 11 ___.___.___ ________  □ Visit 12 ___.___.___ ________ \| □ Blood test  □ Prescription filled  □ Telephone call  □ Training on pump use/disease management  □ Dose titration of PAH treatment  □ Heart catheterization  □ X-ray  □ Coronary angiography  □ 6 min walking test  □ Routine medical check-up  □ ___________ \| __________________  __________________    _________   _________  __________________  _________  _________   _________ \| \| Cardiologist \| □ Public  □ Private \| □ Visit 1 ___.___.___ _________  □ Visit 2 ___.___.___ _________  □ Visit 3 __.___.___ _________  □ Visit 4 ___.___.___ _________  □ Visit 5 ___.___.___ _________  □ Visit 6 ___.___.___ _________  □ Visit 7 ___.___.___ _________  □ Visit 8 ___.___.___ _________  □ Visit 9 ___.___.___ _________  □ Visit 10 ___.___.___ ________  □ Visit 11 ___.___.___ ________  □ Visit 12 ___.___.___ ________ \| □ Blood test  □ Prescription filled  □ Telephone call  □ Training on pump use/disease management  □ Dose titration of PAH treatment  □ Heart catheterization  □ X-ray  □ Coronary angiography  □ 6 min walking test  □ Routine medical check-up  □ ___________ \| __________________  __________________    _________   _________  __________________  _________  _________   _________ \| \| Ambulatory care  in hospital \| □ Public  □ Private \| □ Visit 1 ___.___.___ _________  □ Visit 2 ___.___.___ _________  □ Visit 3 __.___.___ _________  □ Visit 4 ___.___.___ _________  □ Visit 5 ___.___.___ _________  □ Visit 6 ___.___.___ _________  □ Visit 7 ___.___.___ _________  □ Visit 8 ___.___.___ _________  □ Visit 9 ___.___.___ _________  □ Visit 10 ___.___.___ ________  □ Visit 11 ___.___.___ ________  □ Visit 12 ___.___.___ ________ \| □ Blood test  □ Prescription filled  □ Telephone call  □ Dose titration of PAH treatment  □ Heart catheterization  □ X-ray  □ Coronary angiography  □ ___________ \| __________________  __________________   _________  __________________  _________ \| \| Emergency department \| □ Public  □ Private \| □ Visit 1 ___.___.___ _________  □ Visit 2 ___.___.___ _________  □ Visit 3 __.___.___ _________  □ Visit 4 ___.___.___ _________  □ Visit 5 ___.___.___ _________  □ Visit 6 ___.___.___ _________  □ Visit 7 ___.___.___ _________  □ Visit 8 ___.___.___ _________  □ Visit 9 ___.___.___ _________  □ Visit 10 ___.___.___ ________ \| □ Blood test  □ Prescription filled  □ Telephone call  □ Heart catheterization  □ X-ray  □ Coronary angiography  □ ___________ \| __________________  __________________  __________________  _________ \| \| Other (please specify) __________________ \| □ Public  □ Private \| □ Visit 1 ___.___.___ _________  □ Visit 2 ___.___.___ _________  □ Visit 3 __.___.___ _________  □ Visit 4 ___.___.___ _________  □ Visit 5 ___.___.___ _________  □ Visit 6 ___.___.___ _________  □ Visit 7 ___.___.___ _________  □ Visit 8 ___.___.___ _________  □ Visit 9 ___.___.___ _________  □ Visit 10 ___.___.___ ________ \| □ Blood test  □ Prescription filled  □ Telephone call  □ Routine check-up  □ ___________  □ ___________  □ ___________  □ ___________ \| __________________  __________________  ____________________________________ \| \| Other (please specify) __________________ \| □ Public  □ Private \| □ Visit 1 ___.___.___ _________  □ Visit 2 ___.___.___ _________  □ Visit 3 __.___.___ _________  □ Visit 4 ___.___.___ _________ \| □ Blood test  □ Prescription filled  □ Telephone call  □ ___________ \| __________________  __________________ \|   GP: general practitioner; PAH: pulmonary arterial hypertension.  According to the previous Table A, if you have checked that any contact or visit was “Private”, please complete the subsequent Table B. If you have not checked any “private” contact or visit, please move on to question number 2.  Table B. Please indicate the cost (in Euros) per private contact or visit you had. In case you do not remember or have the exact amount, leave it in blank.   \| GP and specialist visits (specialization) \| Number of contacts/visits during the last 6 months \| Costs in € per visit \| \| --- \| --- \| --- \| \| General practitioner \|  \|  \| \| Nurse \|  \|  \| \| Pulmonologist \|  \|  \| \| Cardiologist \|  \|  \| \| Ambulatory care  in hospital \|  \|  \| \| Emergency department \|  \|  \| \| Other (please specify) __________________ \|  \|  \| \| Other (please specify) __________________ \|  \|  \| | | | | |
| 2. During the last 6 months, due to your disease (PAH), were you in hospital or a rehabilitation clinic? | | Yes.……………………………  No……..………………………  If you have checked “Yes” please complete the subsequent table C. If you have  checked “No” please move on to question number 3. | | 1  2 |
| Table C. Please enter the number of activities and dates required.   \| Hospital/  Rehabilitation spells \| Date from – until  (dd-mm-yyyy) \| Date from – until (dd-mm-yyyy) \| Date from – until  (dd-mm-yyyy) \| \| --- \| --- \| --- \| --- \| \| Hospital spells  (dd-mm-yyyy) \| From ___.___.___  Until ___.___.___ \| From ___.___.___  Until ___.___.___ \| From ___.___.___  Until ___.___.___ \| \| Centre  Town \| _________________  _________________ \| _________________  _________________ \| _________________  _________________ \| \| Reason for admittance \| □ Hypoxia  □ Dizziness/syncope  □ Renal impairment  □ Cachexia/anemia  □ Muscle-skeletal pain/atrophy  □ Liver impairment  □ Memory loss/anxiety/depression/frailty  □ Endocrine conditions  □ Pain in the injection site of PAH treatment  □ Endocrine conditions  □ Other  _________________ \| □ Hypoxia  □ Dizziness/syncope  □ Renal impairment  □ Cachexia/anemia  □ Muscle-skeletal pain/atrophy  □ Liver impairment  □ Memory loss/anxiety/depression/frailty  □ Endocrine conditions  □ Pain in the injection site of PAH treatment  □ Endocrine conditions  □ Other  _________________ \| □ Hypoxia  □ Dizziness/syncope  □ Renal impairment  □ Cachexia/anemia  □ Muscle-skeletal pain/atrophy  □ Liver impairment  □ Memory loss/anxiety/depression/frailty  □ Endocrine conditions  □ Pain in the injection site of PAH treatment  □ Endocrine conditions  □ Other  _________________ \| \| Unit of hospital stay \| □ Cardiology  □ Pulmonology  □ Internal Medicine  □ Intensive care unit  □ Emergency box  □ Other  _________________ \| □ Cardiology  □ Pulmonology  □ Internal Medicine  □ Intensive care unit  □ Emergency box  □ Other  _________________ \| □ Cardiology  □ Pulmonology  □ Internal Medicine  □ Intensive care unit  □ Emergency box  □ Other  _________________ \| \| Procedure \| □ Surgery  □ Dose titration of PAH treatment  □ Heart catheterization  □ X-ray  □ Coronary angiography  □ Routine medical check-up  □ Other  _________________ \| □ Surgery  □ Dose titration of PAH treatment  □ Heart catheterization  □ X-ray  □ Coronary angiography  □ Routine medical check-up  □ Other  _________________ \| □ Surgery  □ Dose titration of PAH treatment  □ Heart catheterization  □ X-ray  □ Coronary angiography  □ Routine medical check-up  □ Other  _________________ \| \| Rehabilitation stays  (dd-mm-yyyy) \| From ___.___.___  Until ___.___.___ \| From ___.___.___  Until ___.___.___ \| From ___.___.___  Until ___.___.___ \| | | | | |
| **PARAMEDICAL TREATMENT VISITS** | | | | |
| 3. During the last 6 months, due to your disease, have you regularly been active for prevention or relief of your disease, e.g., by going to rehabilitation groups, alternative practitioner or going to physical therapy?  If so, which type of treatments did you make use of? (e.g., exercise therapy). | | Yes.……………………………  No……..………………………  If you have checked “Yes” please complete the subsequent table D. If you have  checked “No” please move on to question number 4. | | 1  2 |
| Table D. Please enter the number of activities.   \| Activities \| Public or Private? \| Treatments  (please give a short description) \| Number of treatments/visits in the last 6 months \| In case of being Private, indicate the cost (in €), per visit or total cost in the last 6 months \| \| --- \| --- \| --- \| --- \| --- \| \| Physical therapy \| □ Public  □ Private \| 1. __________________  2. __________________ \| __________ \| _________ €  □ Cost per visit  □ Cost per 6 months \| \| Rehabilitation  Occupational therapist \| □ Public  □ Private \| 1. __________________  2. __________________ \| __________ \| _________ €  □ Cost per visit  □ Cost per 6 months \| \| Psychologist \| □ Public  □ Private \| 1. __________________  2. __________________ \| __________ \| _________ €  □ Cost per visit  □ Cost per 6 months \| \| Gym \| □ Public  □ Private \| 1. __________________  2. __________________ \| __________ \| _________ €  □ Cost per visit  □ Cost per 6 months \| \| Alternative practitioner \| □ Public  □ Private \| 1. __________________  2. __________________ \| __________ \| _________ €  □ Cost per visit  □ Cost per 6 months \| \| Social worker \| □ Public  □ Private \| 1. __________________  2. __________________ \| __________ \| _________ €  □ Cost per visit  □ Cost per 6 months \| \| Homeopath/ Naturopath \| □ Public  □ Private \| 1. __________________  2. __________________ \| __________ \| _________ €  □ Cost per visit  □ Cost per 6 months \| \| Acupuncturist \| □ Public  □ Private \| 1. __________________  2. __________________ \| __________ \| _________ €  □ Cost per visit  □ Cost per 6 months \| \| Other (please specify) __________________ \| □ Public  □ Private \| 1. __________________  2. __________________ \| __________ \| _________ €  □ Cost per visit  □ Cost per 6 months \| | | | | |
| **SPECIALIZED EQUIPMENT & DEVICES** | | | | |
| 4. During the last 6 months, did you, e.g., in connection with your disease (PAH) buy yourself medical aids, e.g., wheelchair? Did you even have done modification measures (e.g., build in a stairlift)? Or did you have further expenditures? | | Yes.……………………………  No……..………………………  If you have checked “Yes” please complete the subsequent table E. If you have  checked “No” please move on to question number 5. | | 1  2 |
| Table E. Please enter the names and description.  If you used any equipment but did not pay for it please specify who arranged this for you (e.g., hospital, social services, voluntary sector etc.) at the column “Provided by”.  If you had any adaptations done to your home but did not pay for it, please specify who provided this for you at the column “Provided by”.   \| Medical Aids  (examples) \| Provided by \| Did you have to pay anything? \| Costs in € \| \| --- \| --- \| --- \| --- \| \| □ Wheelchair  □ Special bedding  □ Special mattresses  □ Dehumidifier  □ Medical lift  □ Mechanical ventilation  □ Home oxygen therapy  □ Other _____________________________  □ Other _____________________________ \| ________________________________________________________________________________________________________________  ________________  ________________ \| □ Yes □ No □ N/A  □ Yes □ No □ N/A  □ Yes □ No □ N/A  □ Yes □ No □ N/A  □ Yes □ No □ N/A  □ Yes □ No □ N/A  □ Yes □ No □ N/A  □ Yes □ No □ N/A  □ Yes □ No □ N/A \| _____________________________________________________________________________ \| \| □ Books  □ Videos  □ Other _____________________________ \| ________________________________________________ \| □ Yes □ No □ N/A  □ Yes □ No □ N/A  □ Yes □ No □ N/A \| ____________________________ \| \| □ Private medical care \|  \| □ Yes □ No □ N/A \| _______ \| \| □ Stairlift  □ Ramp  □ Changes to the bathroom/shower  □ Car modifications  □ Other home modifications _____________________________  □ Car modifications _____________________________  □ Other modifications _____________________________  □ Change residence (own)  □ Change residence (new house)  □ Change residence (relative’s house) \| ________________________________________________________________  ________________   ________________   ________________  ________________________________ ________________ \| □ Yes □ No □ N/A  □ Yes □ No □ N/A  □ Yes □ No □ N/A  □ Yes □ No □ N/A  □ Yes □ No □ N/A  □ Yes □ No □ N/A  □ Yes □ No □ N/A  □ Yes □ No □ N/A  □ Yes □ No □ N/A  □ Yes □ No □ N/A \| __________________________________________  ______________  _______  N/A  N/A  N/A \|   N/A: not applicable. | | | | |
| 5. In the following table you may enter all expenses, which you had due to your disease during the last 6 months, which have not been accounted for so far.  After checking this, please move on to question number 6. | | | | |
| \| Further expenses, which have not  been accounted for so far (name) \| Costs in € \| \| --- \| --- \| \| ____________________________________ \| _________ €  □ Unitary cost  □ Cost per 6 months \| \| ____________________________________ \| _________ €  □ Unitary cost  □ Cost per 6 months \| \| ____________________________________ \| _________ €  □ Unitary cost  □ Cost per 6 months \| \| ____________________________________ \| _________ €  □ Unitary cost  □ Cost per 6 months \| | | | | |
| **FORMAL CARE** | | | | |
| 6. During the last 6 months, due to your disease, did you have to hire professional services for homecare? | Yes.……………………………  No……..………………………  If you have checked “Yes” please complete the subsequent table F. If you have  checked “No” please move on to question number 7. | | 1  2 | |
| Table F. Please enter the names and description.   \| Kind of service \| Number of days per week \| Number of hours per day \| Wage per hour (€) \| \| --- \| --- \| --- \| --- \| \| □ Day homecare \|  \|  \|  \| \| □ Night homecare \|  \|  \|  \| \| □ Full time homecare \|  \|  \|  \| \| □ Housekeeping \|  \|  \|  \| \| □ Other  __________________ \|  \|  \|  \| \| □ Other  __________________ \|  \|  \|  \| | | | | |
| **INFORMAL CARE** | | | | |
| 7. During the last 6 months, due to your disease, did you have to accept help from relatives, friends or from professional services for work, which you usually carry out by yourself? For example for housekeeping or for shopping. | Yes.……………………………  No……..………………………  If you have checked “Yes” please complete the subsequent table G. If you have  checked “No” please move on to question number 8. | | 1  2 | |
| Table G. Please insert here the average time in hours of help and the average cost of help per week. In case you could not remember, please insert a question mark.   \| Kind of help \| Average duration  per week \| \| --- \| --- \| \| □ Help from relatives, friends or acquaintances \| ___________ h \| \| □ Home help (e.g., volunteers) \| ___________ h \| \| □ Professional aids (e.g., Red cross) \| ___________ h \| \| □ ___________________ \| ___________ h \| \| □ ___________________ \| ___________ h \| \| □ ___________________ \| ___________ h \| | | | | |
| **PATIENT TRAVEL EXPENSES** | | | | |
| 8. During the last 6 months, due to your disease, how did you travel to go to see your doctor, occupational therapist, or any activity related to your condition?  Once you answered, please complete the Table H below. | Walked………………………  Cycled……………………….  Bus/tramway…………….  Train/metro………..…….  Taxi……………………………  Private car………………….  Motorbike………………...  Hospital car…………….…  Ambulance……………..…  Other (please specify) __________________  __________________ | | 1  2  3  4  5  6  7  8  9  10 | |
| Table H. Please enter the number of kilometers you drove by car, respectively the costs, that aroused from taking other means of transportation during the last 6 months. If you do not know any of these, please enter a question mark.   \| Means of transportation (kind) \| Number of trips (outward and  return voyage equates to one trip) \| Kilometres or cost per ticket (one-way fare) \| \| --- \| --- \| --- \| \| Private car \| ___________ \| ___________ Km \| \| Motorbike \| ___________ \| ___________ Km \| \| Tramway/Bus  (cost of tickets) \| ___________ \| ___________ € \| \| Train  (cost of tickets) \| ___________ \| ___________ € \| \| Taxi  (fares) \| ___________ \| ___________ € \| \|  \| ___________ \| _________ Km/€ \| \|  \| ___________ \| _________ Km/€ \| | | | | |
| 9. If you travelled by private car or motorbike for part or all of the journey and had to pay tolls or parking fees how much did these amounts to? Please write the cost in the box below. Put zero if you did not travel by private car or motorbike at all or did not pay tolls or parking fees. | Expenditure on tolls or parking fees (€) | | ____ | |
| 10. How long did it take to travel from your home/work to the specific location (e.g., general practitioner surgery or hospital clinic)? Please write the number of hours and minutes in the box. | Number of hours Number of minutes | | ________ | |
| 11. In case you had to move into another town different from yours to attend any clinical visit, did you had to cover from your pocket any expenditure (e.g., hotel, meals)? | Yes.……………………………  No……..………………………  If you have checked “Yes” please complete the subsequent table I. If you have  checked “No” please move on to question number 12. | | 1  2 | |
| Table I. Please complete the information.   \| Kind of expenditure \| Number of each expenditure  (e.g., hotel nights, number of meals) \| Total cost (€)  (e.g., per stay) \| \| --- \| --- \| --- \| \| Accomodation  (e.g., hotel) \| ___________ \| ___________ € \| \| Meals  (e.g., restaurants) \| ___________ \| ___________ € \| \| Other extra expenses  __________________ \| ___________ \| ___________ € \|   N/A: not applicable. | | | | |
| **EMPLOYMENT SITUATION** | | | | |
| 12. Are you currently employed? | Yes.……………………………  No……..………………………  If you have checked “Yes” please move on to question number 13. If you have  checked “No” please move on to question number 16. | | 1  2 | |
| 13. If you returned to work in the last 6 months, is it the same employment that you had before your illness? | Yes.……………………………  No……..………………………  N/A…..……………………… | | 1  2  3 | |
| 14. During the last 6 months, have you been on sick leave due to your disease (at home or in hospital)? | Yes.……………………………  No……..………………………  If you have checked “Yes” please complete the subsequent table J. If you have  checked “No” please move to question number 15. | | 1  2 | |
| Table J. Please complete the information.   \| Time you were on  sick leave \| Date from – until (dd-mm-yyyy) \| Date from – until (dd-mm-yyyy) \| Date from – until (dd-mm-yyyy) \| \| --- \| --- \| --- \| --- \| \| Medically certified  absence from work \| From ___.___.___  Until ___.___.___ \| From ___.___.___  Until ___.___.___ \| From ___.___.___  Until ___.___.___ \| \| Reduced hours of  work for gradual  reintegration \| From ___.___.___  Until ___.___.___ \| From ___.___.___  Until ___.___.___ \| From ___.___.___  Until ___.___.___ \| | | | | |
| 15. During the last 6 months, have you seen a doctor or therapist during your working hours? | Yes.……………………………  No……..………………………  If you have checked “Yes” please complete the subsequent table K. If you have  checked “No” please move on to question number 16. | | 1  2 | |
| Table K. Please complete the information.   \| Number of working hours, which  you spent to see a doctor or therapist \| Overall loss of working time \| \| --- \| --- \| \| General practitioner \| ____________________ h \| \| Nurse \| ____________________ h \| \| Cardiologist \| ____________________ h \| \| Pulmonologist \| ____________________ h \| \| Psychologist \| ____________________ h \| \| Ambulatory care in hospital \| ____________________ h \| \| Emergency department \| ____________________ h \| \| Other (please specify) __________________ \| ____________________ h \| \| Other (please specify) __________________ \| ____________________ h \| | | | | |
| 16. Which category describes best your status? | Retired.……………………  Retired on medical grounds.……………………  Unemployed………………  Student.…………….……… | | 1  2  3  4 | |
| 17. During last 6 months have you suffered from income loss due to your illness? | Yes.……………………………  No……..………………………  N/A…..……………………… | | 1  2  3 | |
| 18. Do you have any health-related insurance policy/plan? | Yes.……………………………  No……..………………………  If you have checked “Yes” please complete question 19. If you have checked “No” you have finished the questionnaire. | | 1  2 | |
| 19. Please tell us what it covers by ticking one or more of the following options: | Health care costs (i.e., medical, visits)…………..  Medication………………..  Income protection…….  Any other (please specify) ____________ __________________ | | 1  2  3  4 | |
| 20. Please detail any expenses, visits, etc that have not been included in the previous questions. | | | | |

**References**

- Athanasakis K, Tarantilis F, Tsakalogiannis C, Ollandezos M, Kyriopoulos J; National School of Public Health, Athens, Greece. PCV64: A Cost of Illness Analysis of Pulmonary Arterial Hypertension in Greece. Value in Health. 2016;19:A647.

- Dufour R, Pruett J, Hu N, Lickert C, Stemkowsli S, Tsang Y, et al. Healthcare resource utilization and costs for patients with pulmonary arterial hypertension: real-world documentation of functional class. J Med Econ. 2017 Nov;20(11):1178-86.

- Hagell P, Nordling S, Reimer J, Grabowski M, Persson U. Resource Use and Costs in a Swedish Cohort of Patients with Parkinson’s Disease. Mov Disord. 2002 Nov;17(6):1213-20.

- Keränen T, Kaakkola S, Sotaniemi K, Laulumaa V, Haapaniemi T, Jolma T, et al. Economic burden and quality of life impairment increase with severity of PD. Parkinsonism Relat Disord. 2003 Jan;9(3):163-8.

- Rosenkranz S, Howard LS, Gomberg-Maitland M, Hoeper MM. Systemic Consequences of Pulmonary Hypertension and Right-Sided Heart Failure. Circulation. 2020;141:678-93.

- Schweikert B, Hahmann H, Leidl R. Development and first assessment of a questionnaire for health care utilization and costs for cardiac patients. BMC Health Services Research 2008, 8:187.

- Sikirica M, Iorga SR, Bancroft T, Potash J. The economic burden of pulmonary arterial hypertension (PAH) in the US on payers and patients. BMC Health Serv Res. 2014 Dec;14:676.

- Thompson S, Wordsworth S (on behalf of the UK Working Party on Patient Costs). An annotated cost questionnaire for completion by patients. HERU Discussion Paper No. [03/01]. Available at: [https://www.abdn.ac.uk/heru/documents/BP/HERU_Discussion_paper_03-01.pdf] (Accessed 03/06/2020).
